# Human skeletal muscle macrophages increase following cycle training and are associated with adaptations that may facilitate growth

**DOI:** 10.1038/s41598-018-37187-1

**Published:** 2019-01-30

**Authors:** R. Grace Walton, Kate Kosmac, Jyothi Mula, Christopher S. Fry, Bailey D. Peck, Jason S. Groshong, Brian S. Finlin, Beibei Zhu, Philip A. Kern, Charlotte A. Peterson

**Affiliations:** 1 College of Health Sciences and Center for Muscle Biology, University of Kentucky, Lexington, Kentucky USA; 2 Department of Medicine, Division of Endocrinology, and Barnstable Brown Diabetes and Obesity Center, University of Kentucky, Lexington, Kentucky USA; 3 Deptartment of Nutrition & Metabolism, School of Health Professions, University of Texas Medical Branch at Galveston, Galveston, Texas USA; 4 Department of Health Professions, University of Central Florida, Orlando, Florida USA

## Abstract

Skeletal muscle macrophages participate in repair and regeneration following injury. However, their role in physiological adaptations to exercise is unexplored. We determined whether endurance exercise training (EET) alters macrophage content and characteristics in response to resistance exercise (RE), and whether macrophages are associated with other exercise adaptations. Subjects provided *vastus lateralis* biopsies before and after one bout of RE, after 12 weeks of EET (cycling), and after a final bout of RE. M2 macrophages (CD11b+/CD206+) did not increase with RE, but increased in response to EET (P < 0.01). Increases in M2 macrophages were positively correlated with fiber hypertrophy (r = 0.49) and satellite cells (r = 0.47). M2c macrophages (CD206+/CD163+) also increased following EET (P < 0.001), and were associated with fiber hypertrophy (r = 0.64). Gene expression was quantified using NanoString. Following EET, the change in M2 macrophages was positively associated with changes in HGF, IGF1, and extracellular matrix genes. EET decreased expression of IL6 (P < 0.05), C/EBPβ (P < 0.01), and MuRF (P < 0.05), and increased expression of IL-4 (P < 0.01), TNFα (P < 0.01) and the TWEAK receptor FN14 (P < 0.05). The change in FN14 gene expression was inversely associated with changes in C/EBPβ (r = −0.58) and MuRF (r = −0.46) following EET. In cultured human myotubes, siRNA inhibition of FN14 increased expression of C/EBPβ (P < 0.05) and MuRF (P < 0.05). Our data suggest that macrophages contribute to the muscle response to EET, potentially including modulation of TWEAK-FN14 signaling.

## Introduction

Both endurance and resistance exercise promote maintenance of muscle mass and function^1,2^. Understanding how exercise exerts beneficial effects could provide strategies for mimicking or improving exercise responses. Macrophages participate in muscle repair and regeneration by modulating inflammation, stem cells, cytokines, growth factors, and extracellular matrix. However, their role in the physiological adaptation to exercise is relatively unexplored. Macrophages exhibit phenotypic variability and plasticity, occupying a spectrum from “M1” (inflammatory) to “M2” (anti-inflammatory)^3^.

Macrophages exert effects on myogenic stem cells, satellite cells (SCs), which give rise to myogenic progenitor cells (MPCs). SCs and MPCs express monocyte chemoattractants^4^, and macrophages promote *in vitro* MPC proliferation and differentiation^5,6^. In damaged muscle, M1 macrophages produce inflammatory cytokines (TNFα, IL1β) that signal through canonical NFκB and other pathways to promote SC proliferation^7,8^. In later stages of repair, macrophages shift toward M2 activation, and produce anti-inflammatory cytokines (TGFβ, IL10)^9,10^, driving non-canonical NFκB signaling^11^ and promoting MPC differentiation. Muscle macrophages also produce growth factors, including HGF^12^, which promote SC activation and proliferation^13^. Macrophage depletion impairs recovery from muscle damage caused by contusion^14^, unloading^15^ or neurotoxin delivery^9^.

Macrophages may participate in the regulation of muscle mass by helping to balance catabolic and anabolic signaling. Macrophage-derived inflammatory cytokines, including IL1β, TNFα, and TWEAK, are observed in various disease states, and drive muscle atrophy via canonical NFκB signaling. Canonical NFκB then drives transcription of MuRF, a muscle-specific E3 ubiquitin ligase, leading to protein degradation^7,8,16^. On the other hand, non-canonical NFκB signaling promotes PGC1α production and mitochondrial biogenesis^11,17^. Additionally, M2 macrophages produce IGF1, which supports damage repair, protein synthesis, and maintenance of muscle mass^15,18–20^.

While the macrophage response to muscle injury is well described, their role in adaptation to exercise is largely unstudied. It is clear that the macrophage response to damaging exercise mimics the response to injury, as evidenced by macrophage infiltration following forced lengthening contractions^21^, electrical stimulation^22^, downhill running^23^, and synergist ablation surgery^24^. Macrophage regulation of ECM remodeling is documented in lung^25^, liver^26^, and kidney^27^, where mechanistic studies have shown macrophage regulation of fibroblasts and pericytes. Similar pathways are likely operative in muscle since repair and hypertrophy require ECM remodeling. Accordingly, ECM and M2 macrophage genes are concurrently up-regulated following resistance exercise (RE) and/or endurance exercise training (EET) in humans^28,29^. However, EET caused decreased muscle macrophage content in mice^30^, and did not affect muscle macrophage content in rats^31^.

Muscle macrophage function may be impaired with obesity, aging, and sedentary lifestyle. Our group previously reported higher CD68+ muscle macrophages in obese versus lean humans^32^. We also reported decreased macrophage content, and blunted macrophage response to acute RE, in old versus young men^33^. We and others have shown increased SCs following EET in humans^34–36^. In middle aged women (a subset of this cohort), we have further shown that EET modulates the transcriptional and SC response to RE^37^. We therefore sought to determine whether increased M2 macrophages would be correlated to increased SC content and transcriptional alterations following EET. Since EET alleviates some of the muscle deficits associated with a sedentary lifestyle, we further hypothesized that increasing physical activity via EET would alter the macrophage response to acute RE in humans. Toward this end, we collected muscle from human *vastus lateralis* at baseline, following a single bout of RE, following 12 weeks of EET, and again after a final bout of RE. Immunohistochemistry was used to assess muscle macrophage and SCs and muscle fiber size, and the NanoString nCounter system was used to measure transcription of related genes at each time point.

## Methods

### Human subjects

**Figure 1:**
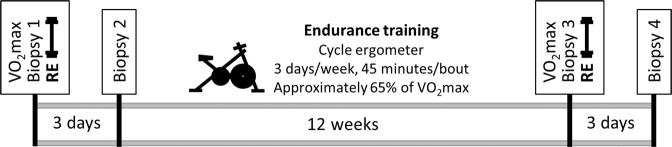
Overview of the study design. At baseline, subjects underwent a *vastus lateralis* biopsy, as well as a fitness assessment by graded exercise test for VO_2_max. Subjects next performed one bout of damaging RE, with a second muscle biopsy taken 3 days later. Subjects then underwent 12 weeks of EET on a cycle ergometer. Exercise intensity was gradually increased and rest periods were gradually decreased so that subjects exercised non-stop for 45 minutes at a heart rate corresponding to approximately 65% of VO_2_max throughout the training period. At the end of the EET protocol, VO_2_max was measured, and a third muscle biopsy was obtained 3 days after the final endurance bout. Participants performed one more bout of RE, with a fourth muscle biopsy obtained 3 days later.

All procedures were performed according to the principles set forth in the Declaration of Helsinki, and approved by the Institutional Review Board of the University of Kentucky. All research participants were informed of the purpose and design of the study, and they provided written consent prior to participation. Exclusion criteria included: smoking, coronary disease, congestive heart failure, chronic inflammatory diseases, or orthopedic problems that could limit the ability to perform the exercise protocols. Participants were sedentary and were asked to maintain consistent dietary and lifestyle habits throughout the study. The cohort includes subjects with a wide range of insulin sensitivity (determined by frequently sampled intravenous glucose tolerance testing, previously described^38^), age, BMI, and aerobic fitness (N = 23, Table 1). Using a small subset (N = 7) of this study cohort, we have previously reported that satellite cell and transcriptional responses to RE were modulated by EET^37^. In this cohort, we have also reported on satellite cell proliferation, muscle fiber hypertrophy (N = 23)^39^ and angiogenesis (N = 26)^38^ in response to EET. In this report, we investigate changes in M2 macrophage response to RE and the effects of EET on that response. We further assess relationships among fiber hypertrophy, muscle macrophages, and gene expression. Figure 1 provides an overview of the study design.

**Table 1:** Characteristics of study participants.

Variable	Mean ± SEM (range)
**Immunohistochemstry using all four biopsy time points**	
N = 14, 78% female	
Age	49.5 ± 3.2 (29–68)
BMI	30.8 ± 1.3 (24.1–39.25)
VO_2_ max	27.5 ± 2.7 (13.8–53.2)
S_I_	3.26 ± 0.53 (0.92–6.81)
**Immunohistochemstry using baseline and post-EET biopsies**	
N = 23, 78% female	
Age	47.5 ± 2.7 (26–68)
BMI	30.8 ± 1.2 (23.3–41.8)
VO_2_ max	27.8 ± 2.0 (13.8–53.2)
S_I_	3.21 ± 0.42 (0.92–7.12)
**NanoString mRNA**	
N = 20, 75% female	
Age	49.8 ± 2.3 (29–64)
BMI	31.5 ± 1.2 (24.3–41.8)
VO_2_ max	27.1 ± 1.8 (13.8–53.2)
S_I_	3.04 ± 0.48 (0.65–7.12)

#### Biopsies

At baseline, we obtained the first of four *vastus lateralis* biopsies using a 5 mm Bergstrom needle with suction. For immunohistochemistry, approximately 100 mg of each biopsy was mounted with fibers perpendicular to a cork using tragacanth gum and snap frozen in liquid nitrogen-cooled isopentane. For isolation of primary myogenic precursor cells (MPCs), approximately 75 mg of tissue was placed in phosphate buffered saline with 1 g/L glucose and penicillin/streptomycin overnight prior to tissue dissociation. For gene expression, the remaining portion of each biopsy was snap-frozen in liquid nitrogen.

#### Graded exercise testing

Subjects next performed maximal graded exercise testing (GXT), with assessment of VO_2_max and integrated electrocardiogram on a calibrated exercise bicycle ergometer. Subjects maintained a pedaling rate of 60–70 rpm, with a workload intensity beginning at 20 watts and increasing by 20 watts every 2 minutes until VO_2_max was achieved. Initial resistance was based on participant fitness. Continuous measures of oxygen consumption and CO_2_ production were obtained using the Vmax 229 system (Viasys Healthcare, Yorba Linda, CA). Respiratory exchange ratio, heart rate, blood pressure, and rate of perceived exertion were recorded in the final 30 seconds of each work watts stage.

#### Resistance exercise

Subjects performed an acute bout of RE using the leg contralateral to the baseline biopsy leg. RE consisted of 5 sets of 8 repetitions of leg extensions at 80% of 1 repetition max, with a sixth set performed to exhaustion. Seventy-two hours after RE, a second muscle biopsy was obtained from the exercised leg.

#### Endurance exercise training (EET)

Participants underwent 12 weeks of EET using a stationary cycle ergometer (Monark 828E Ergometer Upright Bike), and a target intensity corresponding to 65% of VO_2_max and approximately 75–80% of maximum heart rate, as determined by baseline maximal GXT measures. Training intensity was monitored using Polar A3 heart rate monitors (Polar Electro Inc., Woodbury, NY). Subjects were required to exercise three days/week for 45 minutes and were allowed to take intermittent breaks if they were unable to maintain constant exercise for the entire session. Throughout the training period, exercise intensity was gradually increased and rest periods were gradually decreased so that during the eighth through twelfth weeks of training, subjects were able to exercise for 45 minutes consecutively without rest at a heart rate corresponding to 65% of VO_2_max. Average power output per workout was calculated in excel [Power output = (work/time)/6.12].

Following 12 weeks of EET, subjects underwent a third muscle biopsy. In order to detect chronic adaptations to EET, biopsy 3 was performed 72 hours after the last bout of exercise. Next, subjects repeated the RE protocol that was performed at the beginning of the study, and a fourth muscle biopsy was obtained 72 hours later.

### Immunohistochemistry (IHC)

We performed IHC on *vastus lateralis* from 23 subjects, chosen based on the availability and quality of muscle mounts. All staining was performed on 7 µm sections of frozen muscle. Human muscle macrophage IHC has been described in detail and validated in our previous publication^40^. Primary macrophage antibodies include: mouse anti-human CD11b/CR3, clone Bear-1 (Cell Sciences, MON1019-1), mouse anti-human CD163 (Hycult Biotech, HM2157), and goat anti-Human MMR/CD206 (R&D, AF2534). Secondary antibodies include: biotinylated goat anti-mouse IgG1 (Jackson ImmunoResearch, 115-065-205) and biotinylated rabbit anti-goat IgG (Vector Laboratories, BA-5000). Thermo Scientific AlexaFlour systems were used to add fluorescent tags to secondary antibodies. IHC images were obtained using 20× magnification with a Zeiss AxioImager M1 upright microscope and analyzed using Zen Lite software (Carl Zeiss AG, Oberkochen, Germany). Macrophages, fibers, and fiber size were analyzed manually. Macrophages were counted as M2 if they co-stained for CD11b and CD206. For M2 macrophage counting, the smallest section contained 123 cross-sectional fibers and the mean fiber number was 275 ± 15.9 SEM. M2c macrophages were identified by co-staining for CD206 and CD163. For M2c macrophage counting, the smallest section contained 91 cross-sectional fibers and the mean fiber number was 256 ± 22.3 SEM. Macrophage counts were normalized to fiber number. Muscle fiber size was assessed by quantifying cross sectional area (CSA) using IHC against laminin (Abcam, AB14055) to distinguish fiber borders. Satellite cell content was determined using IHC against Pax7 (DSHB), as described in our previous publication^39^.

### Human primary myotube culture, FN14 knock-down, and FN14 overexpression

Human *vastus lateralis* tissue was finely minced in PBS with 1 g/L glucose, dispase II (2.4 U/ml; Roche Applied Science), and collagenase D (1 mg/ml; Sigma-Aldrich). Tissue was then incubated at 37 °C for 60 minutes with gentle rotation, then passed through a 40 μm cell strainer. Cells were washed and pre-plated in in growth medium (HAM’s F-10, 20% fetal bovine serum and 5 ng/ml basic fibroblast growth factor) to allow adherent cells (fibroblasts) to attach. Media containing non-adherent cells (MPCs) was gently moved to a fresh culture dish. MPCs were passaged in growth medium approximately 4 times, allowed to become approximately 90% confluent, then differentiated into myotubes in DMEM with 1 g/L glucose and 2.5% fetal bovine serum. On the fifth day of differentiation, cells were treated with 90 pM scramble RNA or small interfering RNA targeted against FN14 (siFN14) (Invitrogen Stealth RNAi siRNA, ThermoFisher), delivered using Lipofectamine RNAiMAX Reagent (ThermoFisher) according to the manufacturer’s protocol. Forty eight hours after siFN14 treatment, cells were harvested for RNA extraction. For FN14 overexpression, human myotubes were differentiated for 5 days, and then transfected with 500 ng/mL of pCMVScript control (vector) or pCMVScript encoding the full length human FN14 (FN14-FL)^41^. Myotubes were transfected for 24 hours using Lipofectamine 3000 reagent (ThermoFisher), and then harvested for RNA.

### Gene expression

#### RNA extraction

Approximately 30–40 mg of frozen *vastus lateralis* was homogenized in QIAzol Lysis Reagent (QIAGEN, Hilden, Germany, 79306). To obtain RNA from cultured myotubes, cells were washed with PBS and QIAzol was added. Cells were then scraped off of culture dishes and submitted to brief bead homogenization. For both biopsies and cells, RNA was precipitated and washed using the RNeasy kit (QIAGEN, 74104). RNA quantity and quality was assessed using the Agilent 2100 Bioanalyser (Agilent Technologies, Santa Clara, CA).

#### NanoString nCounter

In human muscle biopsies, all genes except TWEAK were measured using the nCounter analysis system (NanoString Technologies, Seattle, WA)^42–44^. The nCounter system allowed us to design a hypothesis-driven custom probe set containing 6 housekeeping genes and 102 genes of interest related to muscle hypertrophy, extracellular matrix remodeling, macrophages, and inflammation^37^. The custom code set was then hybridized with 100 ng of RNA from each biopsy. Gene expression was normalized to the geometric mean of six housekeeping genes (β-actin, Cyclophilin A, Cyclophilin B, TATA binding protein, Tubulin-β, and Ubiquitin C), and the mean of eight negative controls was subtracted. The data are presented as normalized counts. Eight genes in the code set were not detectable, leaving us with 94 genes available for analysis. Subjects used for NanoString analysis were the first 20 subjects who completed the study and had sufficient RNA quality (RIN ≥ 7.0) at each time point.

#### Real-time PCR

Real-time PCR was used to measure changes in gene expression in cultured human myotubes following FN14 knock-down, as well as TWEAK expression in muscle biopsies (TWEAK was not included in the NanoString code set). RNA was reverse transcribed with SuperScript™ IV VILO™ (ThermoFisher) and quantitative real-time rtPCR was performed using PowerUp™ SYBR™ Green Real-Time PCR Master Mix (ThermoFisher). Gene expression was calculated using the 2^ΔΔCt method, with genes of interest normalized against the geometric mean of three housekeeping genes (18S RNA, β2 microglobulin (B2M), and phosphoglycerate kinase (PGK)). Primer sequences are given in Supplementary Table 1.

### Statistics

All data are expressed as mean ± SEM. Significance was predetermined to be P ≤ 0.05. Exact P values for biologically relevant trends are reported. 2-way ANOVA was used to determine whether RE or EET affected skeletal muscle macrophage content, with RE, EET, and RE x EET included in the model as independent variables. Paired t-tests were used to determine the effects of endurance training (biopsy 1 versus biopsy 3) on gene expression and macrophages/fiber. Paired t-tests were also used to determine the transcriptional effects of FN14 knock-down and overexpression in cultured myotubes. Linear regressions employed the Pearson product-moment correlation coefficient when the two continuous variables were normally distributed (including all figures depicting regressions). Because we designed a hypothesis-driven custom code set, NanoString gene expression analyses were not corrected for multiple comparisons. Statistical analyses were performed with JMP v. 12 (SAS Institute, Cary, NC).

## Results

Baseline subject characteristics are provided in Table 1. The study consisted of mostly women with a broad range in age (29–68 years), BMI (23.3–41.8) and fitness (VO_2_max 13.8–53.2). Menopause status and medications are provided in Supplementary Table 2. All four biopsies were available for macrophage immunohistochemistry (IHC) from 14 subjects, with baseline and post-EET biopsies available for 23 of the 26 subjects who completed the trial. RNA was isolated for NanoString gene expression analyses on biopsies from baseline and post EET from 20 subjects. Both IHC and NanoString results were available at baseline and post EET on biopsies from 17 subjects. There were no statistically significant differences between the subgroups of subjects used for differing analyses (Table 1).

### M2 macrophages increase following EET and are associated with fiber hypertrophy and satellite cells

**Figure 2:**
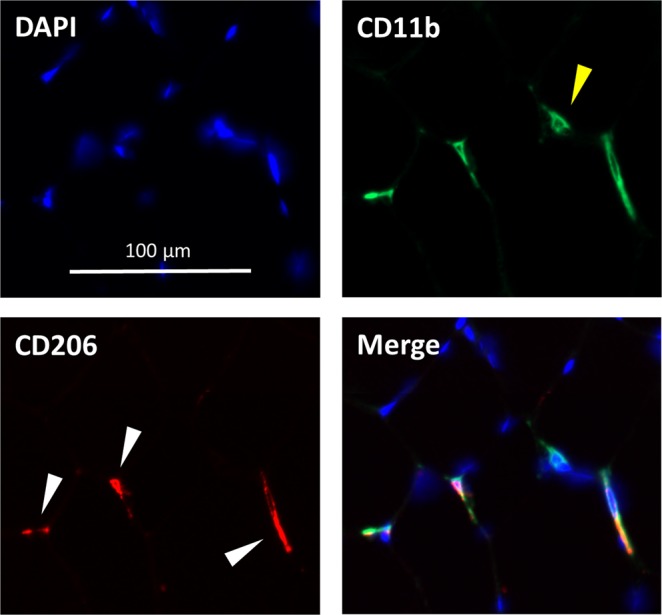
Representative skeletal muscle macrophage immunohistochemistry. M2 macrophages were identified via co-stain for CD11b and CD206. Blue DAPI stain identifies nuclei. Green indicates CD11b, used as a pan-macrophage marker. Yellow carrot indicates a CD11b+/CD206- macrophage. Red indicates CD206, used to identify M2 macrophages. White carrots indicate cells that express both markers (CD11b+/CD206+). A merged image shows CD11b, CD206, and DAPI.

**Figure 3:**
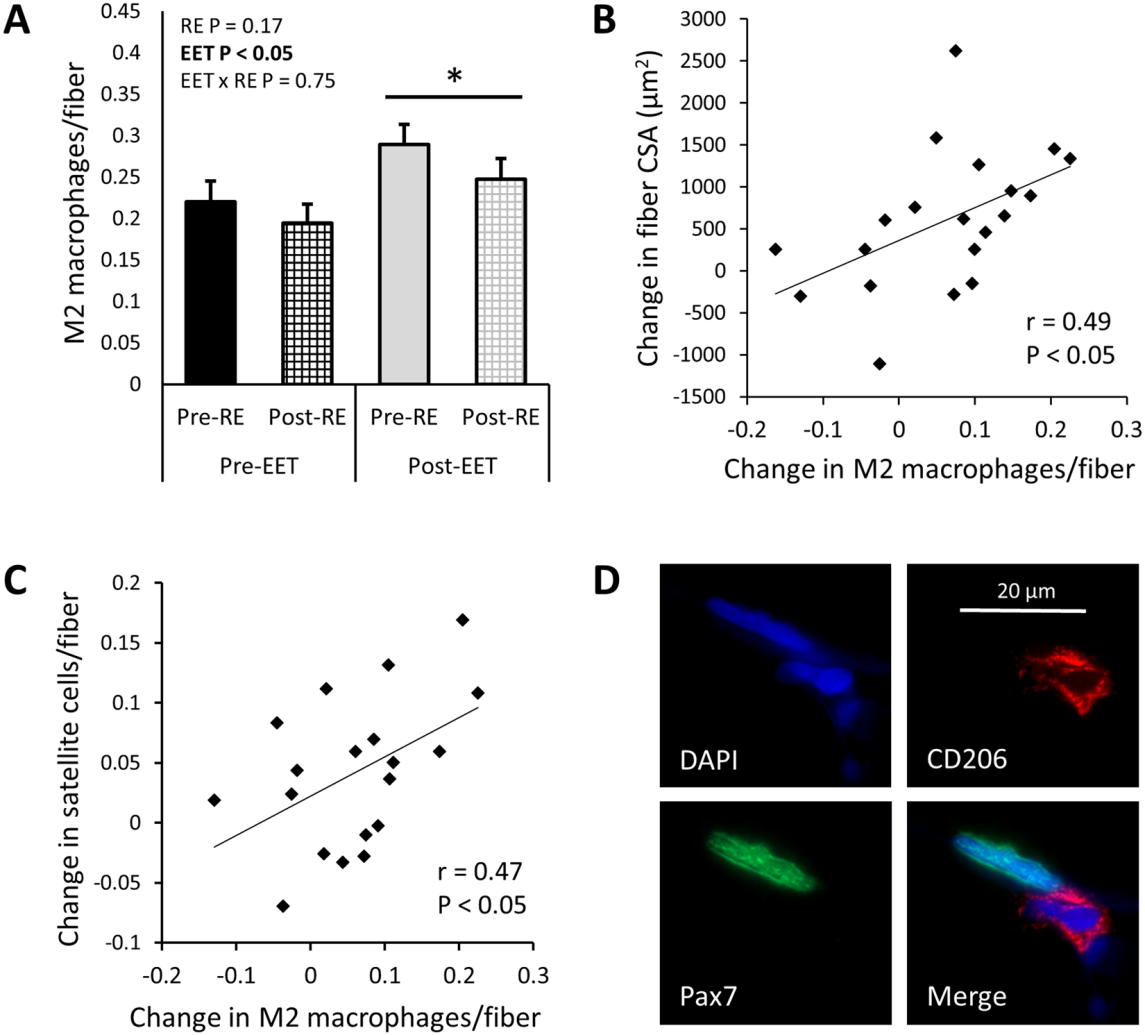
In human *vastus lateralis*, macrophages increase following 12 weeks EET and are associated with muscle fiber hypertrophy. (**A**) M2 (CD11b+/CD206+) macrophages do not increase 3 days after a single bout of RE. However, M2 macrophages are increased following EET (P < 0.05) and EET does not modulate the macrophage response 3 days after a final bout of RE (RE x EET *NS*, N = 14, 2-way ANOVA). (**B**) Following EET, the increase in macrophages per fiber is associated with muscle fiber hypertrophy, assessed by change in fiber cross-sectional area (CSA) (r = 0.49, P < 0.05, N = 20). (**C**) The change in M2 macrophages per fiber is also positively associated with increased satellite cells per fiber (assessed by Pax7 IHC) following EET (r = 0.47, P < 0.05, N = 18). (**D**) M2 macrophages are sometimes observed in close proximity to Pax7+ satellite cells, as in this 100x z-stack image.

Since macrophages are known to participate in the response to muscle injury^45,46^, we hypothesized that a single bout of high intensity RE in sedentary humans would induce muscle macrophage infiltration within 3 days. We further hypothesized that EET in the sedentary participants would modify the subsequent response to RE. We therefore performed IHC staining for CD11b (a pan macrophage marker) and CD206 (an M2 macrophage marker) on muscle biopsies taken at baseline, 3 days after RE, 3 days after the last EET session, and 3 days after a second bout of RE (Fig. 1 shows an overview of the study design). Representative macrophage staining is shown in Fig. 2. The M2 (CD11b+/CD206+) macrophage response to the exercise protocols is shown in Fig. 3A. Contrary to our original hypothesis and differing from our findings in a previous cohort^33^, macrophages/fiber were not increased 3 days after performing RE (leg extensions) to exhaustion (RE P = 0.17; Fig. 3A). However, 12 weeks cycle ergometer EET elicited a significant increase in M2 macrophages/fiber (EET P < 0.05; Fig. 3A), and EET did not alter macrophage numbers in response to a final bout of RE (RE x EET P = 0.75; Fig. 3A). We were able to perform IHC for M2 macrophages in 9 additional subjects with available biopsies from baseline and following EET, which confirmed the significant increase in M2 macrophages following EET (P < 0.01, N = 23; Supplementary Fig. 1). Finally, we counted cells that were CD11b+/CD206−. In human skeletal muscle, these cells are rare and may constitute an M1-like population. However we did not observe sufficient numbers of these cells to perform statistical analyses.

In this cohort, we previously reported muscle fiber hypertrophy (assessed by fiber cross -sectional area (CSA) using IHC for laminin) and increased satellite cell content (assessed by IHC for Pax7) following EET^39^. Because macrophages are known to participate in tissue remodeling^47^, we investigated whether the EET-induced increase in macrophages was associated with increased fiber size and satellite cells. Indeed, the change in M2 macrophages/fiber was positively associated with the change in fiber CSA following EET (r = 0.49, P < 0.05; Fig. 3B). Additionally, the change in M2 macrophages/fiber was also positively associated with the change in satellite cells/fiber resulting from EET (r = 0.47, P < 0.05; Fig. 3C). On six tissue sections, we performed IHC for both Pax7 and CD206, and we observed several instances of M2 macrophages in close proximity to satellite cells (Fig. 3D). Although most participants showed gains in average power output per 45 minute workout (watts) following EET (P < 0.01; Supplementary Fig. 2), there was no relationship between gains in average power and increased muscle macrophage content. Similarly, most participants showed increased VO_2_max following EET (P < 0.01; Supplementary Fig. 3), but changes in VO_2_max were not correlated with changes in muscle macrophage content. Moreover, there were no relationships among age, BMI, or change in BMI and baseline M2 macrophages or change in M2 macrophages with EET.

#### Changes in M2 macrophages are associated with changes in gene expression in response to EET

**Figure 4:**
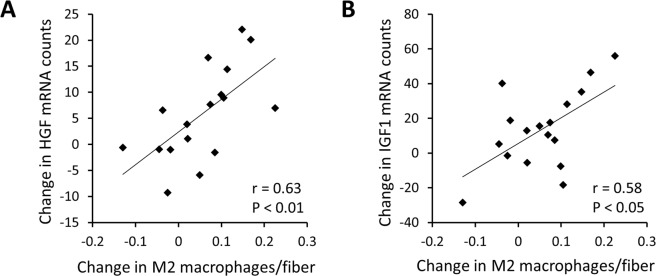
Following EET, the change in M2 macrophages per fiber is positively associated with gene expression changes in the hypertrophic growth factors (**A**). HGF (r = 0.63, P < 0.01), and (**B**). IGF1 (r = 0.58, P < 0.05) in human *vastus lateralis*. Gene expression changes were measured using the NanoString nCounter system. P values are not adjusted for multiple comparisons. N = 17.

Using a hypothesis-driven, custom-designed NanoString code set, we measured expression of genes related to macrophages/inflammation, growth and ECM expansion, at baseline and following EET. Changes in M2 macrophage abundance, assessed by IHC, were significantly correlated to changes in CD11b (ITGAM gene) and CD206 (MRC1 gene) mRNA counts, as well as other macrophage markers (Table 2). Consistent with the observation that macrophages are associated with fiber hypertrophy and satellite cell number, the training-induced increase in macrophages was also positively associated with increased transcription of two growth factor genes: HGF, which is up-regulated during surgical overload-induced hypertrophy^48,49^ (r = 0.63, P < 0.01; Fig. 4A), and IGF1 (isoform 4; class IA), whose splice variants are known to drive various stages of the hypertrophic response to exercise^50,51^ (r = 0.58, P < 0.05; Fig. 4B). Furthermore, the change in M2 macrophages following EET was positively associated with changes in numerous extracellular matrix related genes (Table 2). Other growth, ECM and inflammation genes that were not correlated to changes in macrophages are shown in Supplementary Table 3. Several genes whose changes were significantly correlated to the change in M2 macrophage number were also significantly altered by EET, including: HGF, IGF1, CD11b (ITGAM), SPARC (osteonectin), LOX (lysyl oxidase), COL6A1, COL5A1, and SERPINE1 (PAI-1) (See Table 2 and Supplementary Table 4).

**Table 2:** Correlation between changes in M2 macrophages/fiber and changes in ECM and inflammation gene expression following EET.

Gene name	Alternate name	r	P
**Inflammation**			
CCL18	C-C motif chemokine ligand 18	0.73	0.001**
ITGAX	Integrin subunit alpha X, CD11c	0.58	0.014*
ITGAM	Integrin subunit alpha M, CD11b	0.58	0.014*
CCL5	C-C motif chemokine ligand 5, RANTES	0.56	0.019*
CD68	CD68 molecule	0.56	0.019*
HMOX1	Heme oxygenase 1	0.51	0.035*
MRC1	Mannose receptor C-type 1, CD206	0.49	0.045*
**Extracellular matrix**			
MMP14	Matrix metallopeptidase 14	0.69	0.002*
SERPINE1	Plasminogen activator inhibitor-1, PAI-1	0.64	0.006*
SPARC	Osteonectin	0.63	0.007*
ELN	Elastin	0.56	0.018*
COL5A1	Collagen type V α-1 chain	0.56	0.021*
COL6A1	Collagen type VI α-1 chain	0.54	0.026*
TGFB1	Transforming growth factor β-1	0.50	0.040*
LOX	Lysyl oxidase	0.49	0.047*

M2 (CD11b+/CD206+) macrophages were assessed by IHC. Gene expression was quantified using a custom-designed NanoString nCounter system code set. r = Pearson correlation coefficient. **P < 0.01, *P < 0.05. P values are not adjusted for multiple comparisons. N = 20.

#### CD163+ M2c macrophages increase following EET and are associated with fiber hypertrophy

**Figure 5:**
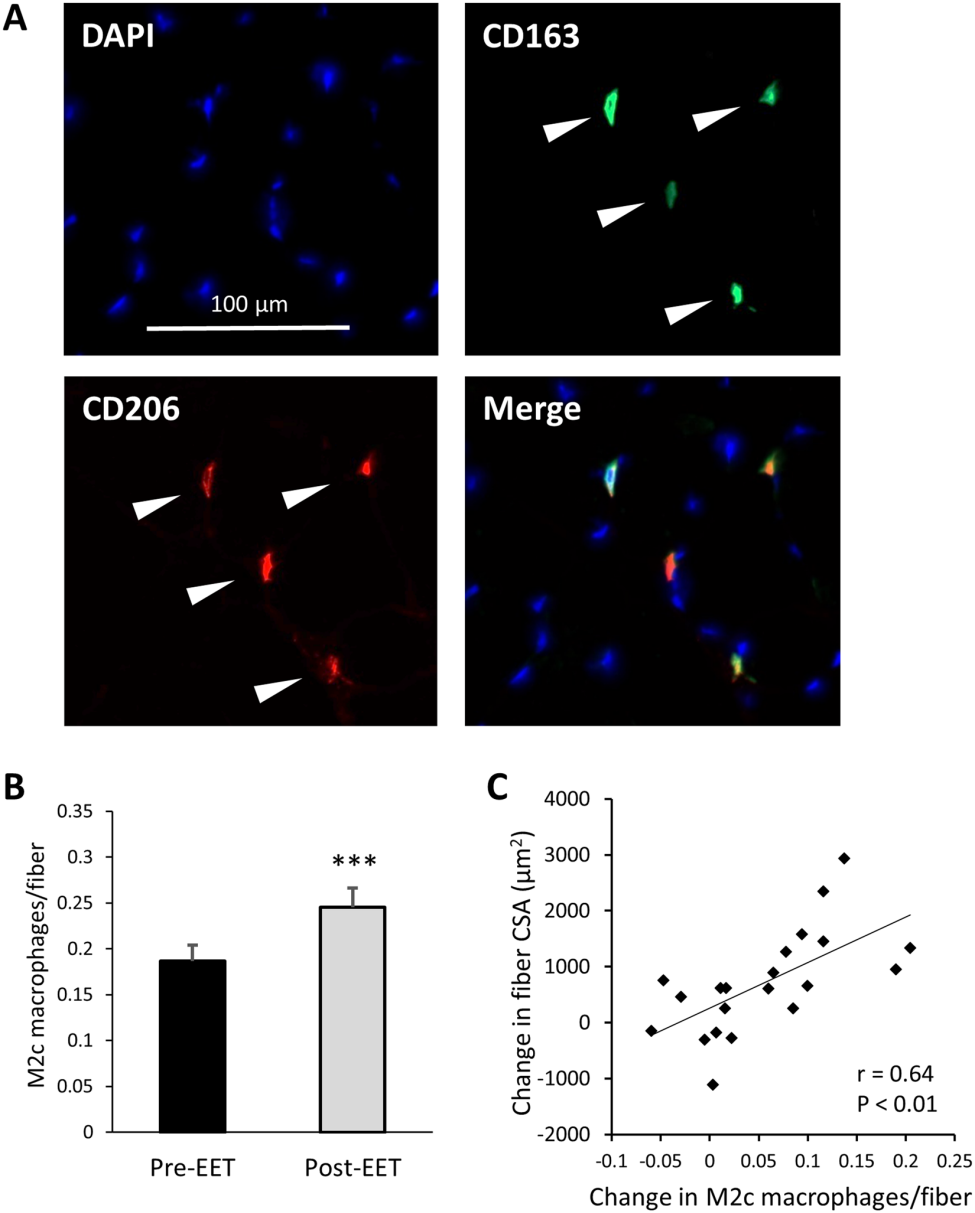
In human *vastus lateralis*, M2c (CD206+/CD163+) macrophages increase following 12 weeks EET and are associated with muscle fiber hypertrophy. (**A**) Representative skeletal muscle M2c macrophage immunohistochemistry: Nuclei were stained with DAPI (blue). CD163 stain (green) was used as a specific M2c marker, while CD206 (red) was used as a pan M2 marker. A merged image shows M2c macrophages that co-stain for CD206 and CD163. White carrots indicate CD206+/CD163+ M2c macrophages. (**B**) EET results in increased M2c macrophages per fiber (P < 0.001, paired t-test), and (**C**) the increase in M2c macrophages per fiber is associated with muscle fiber hypertrophy, as assessed by change in fiber cross-sectional area (CSA, µm^2^) (r = 0.64, P < 0.01). N = 20.

M2 macrophages are increasingly understood to include a functionally diverse set of cells, many of which express CD163, and are known as M2c macrophages. M2c macrophages are believed to be involved in extracellular matrix remodeling, since they are stimulated by TGFβ and they produce TGFβ^52^. We therefore sought to determine whether EET increases CD163+ M2c macrophages. Representative CD163 staining is shown in Fig. 5A. CD163 was expressed on 67.1% (±4.9% SEM) of CD206+ macrophages prior to training, and on 70.0% (±4.7% SEM) after training (not significant). Although EET did not influence the ratio of CD163+ to total M2 macrophages, it resulted in a highly significant increase in M2c macrophages per fiber (P < 0.001; Fig. 5B). As with total CD206+ M2 macrophages, the increase in M2c macrophages was tightly correlated to muscle fiber hypertrophy following EET (r = 0.64, P < 0.01; Fig. 5C). However, change in M2c macrophages was not associated with change in satellite cells, HGF mRNA, or IGF1 mRNA. Whereas we observed numerous associations between M2 macrophages and gene expression, changes in M2c macrophages were only associated with changes in 6 genes: TIMP2 (r = 0.63, P < 0.01), MMP2 (r = 0.54, P < 0.05), TNFα (r = 0.55, P < 0.05), CD68 (r = 0.54, P < 0.05), and tissue factor (F3 gene) (r = 0.53, P < 0.05). Lastly, neither baseline M2c macrophages nor change in M2c macrophages were associated with age, BMI, VO_2_max, or change in BMI or VO_2_max.

#### EET alters inflammation-related gene expression

**Figure 6:**
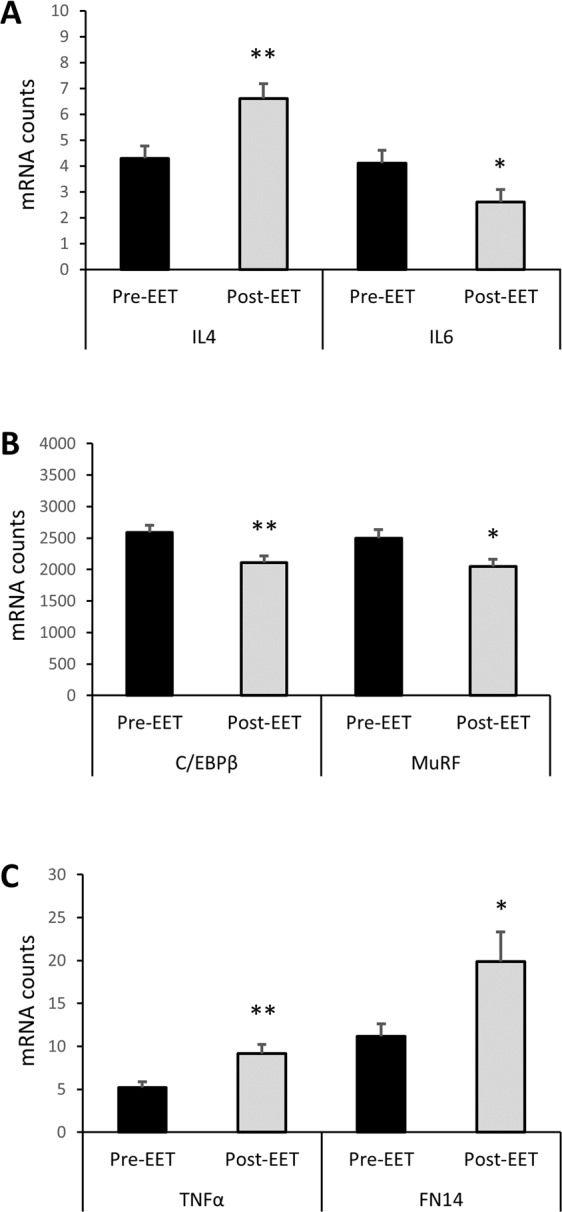
Effect of 12 weeks of EET on inflammation-related gene expression in human *vastus lateralis*. Gene expression was measured using the NanoString nCounter system. (**A**) The anti-inflammatory cytokine IL4 is increased (P < 0.01), while the pleiotropic cytokine IL6 is decreased (P < 0.05) following EET. (**B**) EET results in decreased expression of the NFκB target genes C/EBPβ (P < 0.01) and MuRF (P < 0.05). (**C**) EET causes increased expression of TNFα (P < 0.01) and the TNF receptor super family member FN14 (P < 0.05). Paired t-tests. P values are not adjusted for multiple comparisons. N = 20.

Expression of several inflammatory and anti-inflammatory genes were altered with EET, but were not directly correlated to changes in M2 or M2c macrophage number. The anti-inflammatory cytokine IL4 exhibited increased gene expression following EET (P < 0.01; Fig. 6A), whereas the inflammatory cytokine IL6 exhibited decreased gene expression after training (P < 0.05; Fig. 6A). Moreover, EET led to decreased expression of other known NFκB interacting genes, including the transcription factor C/EBPβ (P < 0.01; Fig. 6B) and the muscle specific E3 ubiquitin ligase MuRF (P < 0.05; Fig. 6B). C/EBPβ can heterodimerize with NFκB subunits to either antagonize or synergistically promote gene transactivation^53–55^, while MuRF is a known NFκB p50/65 target gene in skeletal muscle^56–58^. In spite of reduced expression of IL6, C/EBPβ, and MuRF, other known NFκB interacting genes were induced by EET, including TNFα (P < 0.01; Fig. 6C), which participates in MPC activation^59,60^, and FN14 (P < 0.05; Fig. 6C), a member of the TNF receptor superfamily. Supplementary Table 4 shows the effect of EET on expression of other inflammation and macrophage related genes. We were unable to determine whether IL10 mRNA was affected by EET because it was below the detectable limit of our NanoString code set.

#### FN14 expression is inversely related to C/EBPβ and MuRF gene expression

**Figure 7:**
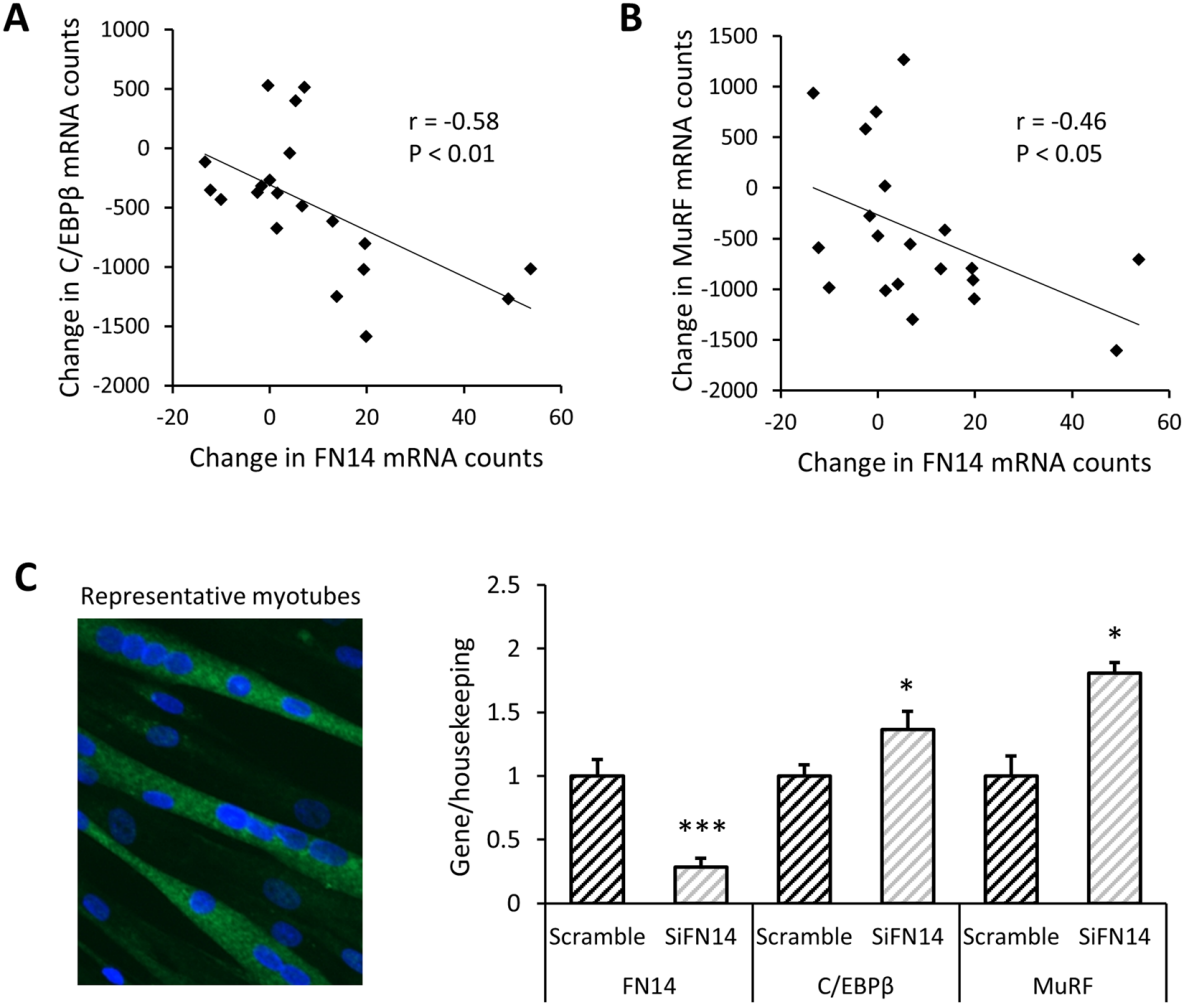
FN14 gene expression is inversely correlated to expression of C/EBPβ and MuRF. In human *vastus lateralis*, change in FN14 expression is inversely correlated to change in (**A**). C/EBPβ expression (r = −0.58, P < 0.01), and (**B**) MuRF expression (r = −0.46, P < 0.05) resulting from 12 weeks endurance exercise training. P values are not adjusted for multiple comparisons. N = 20. (**C**) In cultured primary human myotubes, siRNA was used to knock down FN14 (siFN14), leading to a 71% decrease in FN14 expression (P < 0.001), together with increased expression of C/EBPβ (P < 0.05) and MuRF (P < 0.05) compared to scramble control cells. Paired t-tests were used to analyze data using 2 wells of each treatment in 2 individual cell lines and 1 well of each treatment in a third cell line (N = 5 data points per condition).

Fn14 and its endogenous ligand TWEAK are known to exert paradoxical effects in skeletal muscle^61^. Furthermore, FN14 transcription has been shown to increase acutely following exercise^62,63^ and to be associated with fiber hypertrophy^64^. In our cohort, EET increased FN14 gene expression, but did not affect TWEAK gene expression (Supplementary Fig. 4). Since FN14 signaling can activate both canonical and non-canonical NFκB pathways^65^, we hypothesized that the change in FN14 expression would relate to changes in other inflammation related genes that were modulated by EET. After training, the change in FN14 expression was not correlated to changes in IL4, IL6, or TNFα. However, the training-induced change in FN14 was positively associated with the change in myogenin (r = 0.58, P < 0.05; Supplementary Fig. 5), a transcription factor that is required for muscle differentiation and has been reported to be inhibited by the NFκB p65 subunit^11^. Moreover, the EET-induced increase in FN14 mRNA was inversely correlated to changes in C/EBPβ (r = −0.58, P < 0.01; Fig. 7A) and MuRF (r = −0.46, P < 0.05; Fig. 7B). Given these observations, we hypothesized that when TWEAK remains constant, FN14 may inhibit transcription of C/EBPβ and MuRF. To test this hypothesis, we delivered small interfering RNA against FN14 (siFN14) to cultured primary human myotubes. Forty eight hours after siFN14 delivery, expression of FN14 was reduced by 71% versus scramble control (P < 0.001; Fig. 7C). TWEAK gene expression is readily detectible in human myotubes, with an average Ct of 26.1 ± 0.16 SEM, or approximately 15% of the level of FN14 in scramble cells. Neither TWEAK nor myogenin gene expression were affected by FN14 knock-down. However, siFN14 caused increased gene expression of both C/EBPβ (P < 0.05; Fig. 7C) and MuRF (P < 0.05; Fig. 7C). However, overexpression of FN14 in human myotubes did not have the opposite effect. Transfection with FN14-FL induced a 35.5-fold (±3.45 SEM) increase in FN14 gene expression (P < 0.001), without affecting expression of C/EBPβ or MuRF (Supplementary Fig. 6).

## Discussion

We tested the hypothesis that EET would enhance the macrophage response to an acute bout of RE. We were surprised to observe that muscle macrophage content was increased following EET, but not RE. To our knowledge, we are the first to show that human muscle M2 and M2c macrophages increase following 12 weeks EET. In rodent studies, EET appears to reduce or have no effect on muscle macrophage content^30,31^. Our results in humans may differ from those observed in rodents because each of our endurance workouts contained some resistance stimulus and led to increased fiber CSA. Furthermore, we incrementally increased the cycle ergometer resistance throughout the study, contributing to increased average power output/workout as the study progressed.

The EET-elicited increase in M2 macrophage content was associated with increased satellite cell content. HGF and IGF1 may mediate the satellite cell response, as mRNAs encoding both growth factors are increased in proportion to M2 macrophages following EET, consistent with the idea that macrophages are an important source of these growth factors and play an active role in modulating the satellite cell niche^12,18^. Accordingly, we observed instances of macrophages in close proximity to satellite cells. This is consistent with reports indicating that macrophage-mediated SC activation requires proximal positioning^66^, and that the two cell types may share areas where intercellular space is less than 20 nm wide, termed “tight surface appositions”^67^.

Changes in genes associated with ECM remodeling were also positively correlated to changes in M2 macrophages with training, which is consistent with previous gene expression reports^28,29^ and is also consistent with the supposition that macrophages are involved in the hypertrophic response to EET and contribute to ECM allostasis. We did not perform statistical corrections for multiple tests because our NanoString code set was specifically designed to augment our overarching hypothesis that changes in macrophages would be associated with changes in growth-related, ECM, and inflammatory genes. Therefore, all correlational data should be interpreted as hypothesis generating rather than causal or mechanistic in the context of whole animal exercise responses.

EET caused changes in inflammation-related gene expression, and some of these changes were not directly correlated to changes in macrophage abundance. Nonetheless, increased expression of IL4, along with decreased expression of IL6, C/EBPβ, and MuRF, is consistent with a shift toward anti-inflammatory, pro-hypertrophic signaling following EET, and may result from changes in macrophage function. However, we were intrigued by the observation that EET resulted in up-regulation of TNFα and FN14, the receptor for TWEAK. Both TNFα and TWEAK exert pleiotropic effects on skeletal muscle. While TNFα is thought to promote satellite cell/MPC proliferation^59,60^, TWEAK has been shown to either promote^68,69^ or inhibit^70^ satellite cell/MPC proliferation. When chronically elevated, both TNFα and TWEAK drive canonical NFκB p50/p65-mediated protein catabolism and muscle loss^16,59,65,71–73^. MuRF is a known transcriptional target of p50/p65^58,74^, and C/EBPβ and p50/p65 can synergistically transactivate numerous gene promoters^55^. We therefore hypothesize that C/EBPβ may act synergistically with p50/p65 to drive MuRF transcription. Indeed, the MuRF promoter contains 8 predicted p65 response elements and 4 predicted CCAAT (C/EBPβ interacting) motifs within 2000 bp of its transcription start site^75,76^.

FN14-TWEAK signaling is primarily regulated through FN14 abundance, as TWEAK did not change with EET. Although FN14 has primarily been studied within the context of muscle damage and regeneration^61^, FN14 transcription has been associated with hypertrophic stimuli. FN14 is acutely elevated following a single bout of either endurance or resistance exercise^62,63^. It is associated with type II fiber hypertrophy following taper from training^64^. Furthermore, FN14 was induced approximately 500-fold in an individual champion sprint runner following a single bout of resistance exercise^77^. These associations between FN14 and hypertrophic stimuli are consistent with our observation that transcription of C/EBPβ and MuRF genes are inversely associated with FN14 in our training cohort, and that C/EBPβ and MuRF mRNAs are increased following FN14 knock-down in primary human myotubes. The apparent disconnect between ligand (TWEAK) and receptor (FN14) effects may be explained by TWEAK-independent effects of FN14. For example, FN14 has been shown to promote myogenin expression regardless of TWEAK levels in C2C12 cells^78^. Additionally, TWEAK expression is higher in macrophages compared to other cell types^79^ and increased levels of membrane bound TWEAK relative to soluble TWEAK promotes canonical NFκB signaling^80^. Thus, relatively low levels of TWEAK in our human myotube cultures may favor non-canonical NFκB signaling via FN14 and the subsequent suppression of C/EBPβ and MuRF transcription. Furthermore, overexpression of FN14 in cultured myotubes did not affect C/EBPβ or MuRF gene expression, indicating that basal levels of TWEAK-independent FN14 signaling may be sufficient to suppress C/EBPβ and MuRF transcription.

Here we report a highly significant increase in CD163+ M2c macrophages following EET. CD163 is highly expressed on tissue resident macrophages^81^ and is associated with the M2c macrophage transcriptional program *in vitro*^82^. Membrane bound CD163 decreases TWEAK bioactivity by binding and internalizing it^83^, while soluble CD163 functions as a decoy receptor for TWEAK^84,85^. We hypothesize that when CD163 is low and TWEAK-FN14 interactions high, canonical NFκB signaling may dominate, favoring C/EBPβ and MuRF transcription and atrophy. Conversely, when CD163 is abundant and TWEAK-FN14 interactions are decreased, C/EBPβ and MuRF transcription may be suppressed, and hypertrophic pathways predominate. We did not expect, nor did we observe, a correlation between increased M2c macrophage content and increased FN14 expression since FN14 is expressed by myofibers and participates in both hypertrophic and atrophic signaling. Thus, optimal hypertrophic responses may include alterations in cytokine signaling, FN14 inhibition of C/EBPβ and MuRF, which may be augmented by increased CD163 abundance, and increased M2 macrophage signaling via HGF and IGF1.

In the current cohort, we were surprised to observe no macrophage response to RE. This may be due to the heterogeneity of the cohort, which included individuals with a broad range of age and metabolic health. We previously reported a macrophage response 3 days after RE in young healthy men, which was reduced in older men^33^. Nonetheless, our current results might also be explained by the timing of our biopsy collection. While we performed *vastus lateralis* biopsies 72 hours after RE, an elegant study by Mackey and Kjaer performed biopsies 7 days after a highly damaging electrical stimulation protocol and reported dramatic macrophage infiltration in humans^21^. We also suspect that changes in macrophage morphology could account for inconsistent detection before and after RE; “amoeboid” (contracted, spherical) macrophages have been associated with inflammatory signaling while “ramified” (flattened, with multiple processes) macrophages have been associated with quiescence, surveillance, and/or anti-inflammatory signaling^86,87^. If muscle macrophages are more amoeboid following RE, they may be less likely to appear in a 7 µm tissue slice and therefore more difficult to quantify.

Taken together, these results indicate that in the absence of severe tissue damage, muscle resident macrophages are more polarized toward M2 than previously appreciated. Following endurance exercise training, M2 macrophages are associated with fiber hypertrophy and satellite cell accumulation, and may reduce inflammation, potentially via suppression of TWEAK signaling. Further elucidation of the beneficial functions of skeletal muscle macrophages following exercise could lead to novel approaches to overcome muscular deficits observed in obesity, aging, and other pathological conditions.

## Supplementary information

Supplementary Figures and Tables
