# Supplementary material for: Human skeletal muscle macrophages increase following cycle training and are associated with adaptations that may facilitate growth
Source: Sci Rep. 2019 Jan 30;9:969. doi: 10.1038/s41598-018-37187-1 (PMC6353900; doi:10.1038/s41598-018-37187-1)
Supplement: Supplementary file 1 — Supplementary Figures and Tables [file 41598_2018_37187_MOESM1_ESM.pdf]

Human skeletal muscle macrophages increase following cycle training and are associated with adaptations that may facilitate growth

R. Grace Walton, Kate Kosmac, Jyothi Mula, Christopher S. Fry, Bailey D. Peck, Jason S. Groshong, Brian S. Finlin, Beibei Zhu, Philip A. Kern, and Charlotte A. Peterson

Supplementary Materials

S1

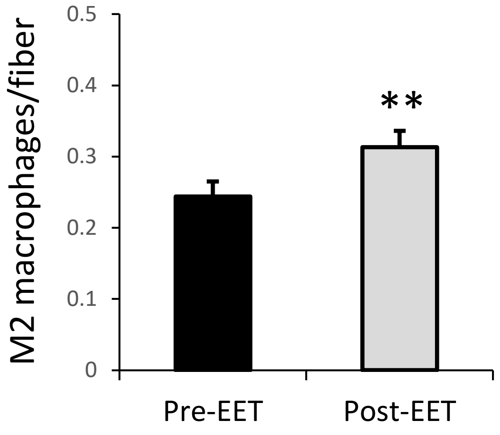

Supplementary figure 1. Skeletal muscle M2 (CD11b+/CD206+) macrophages increase following 12 weeks EET (P < 0.01, paired t-test). N=23.

S2

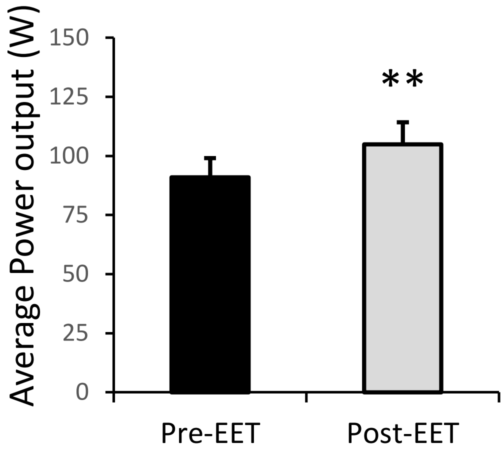

Supplementary figure 2. Cycle ergometer power output increases with endurance exercise training. (P <0.01, paired t-test). N=22.

S3

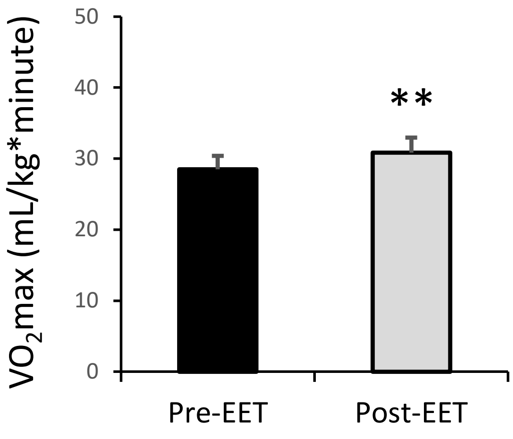

Supplementary figure 3. VO2max increases following endurance exercise training. (P <0.01, paired t-test). N=23.

S4

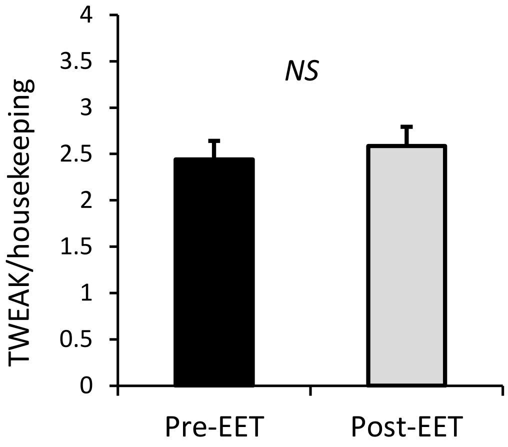

Supplementary figure 4. In human *vastus lateralis* TWEAK expression, assessed by rt-PCR, is not affected by endurance exercise training. Paired t-test *NS*. N=19.

S5

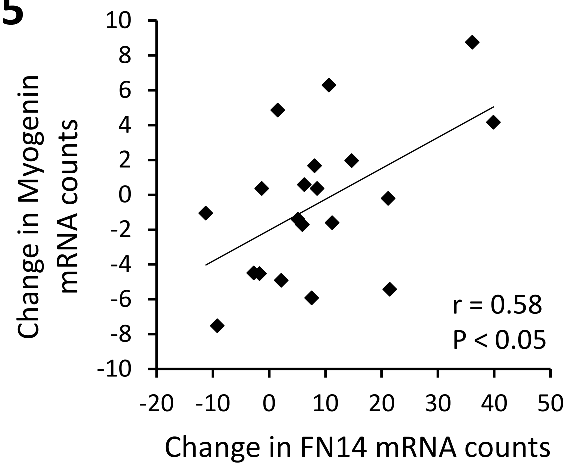

Supplementary figure 5. Following EET, the change in FN14 gene expression is positively associated with the change in myogenin gene expression ($r = 0.58$, $P < 0.05$). N=20.

S6

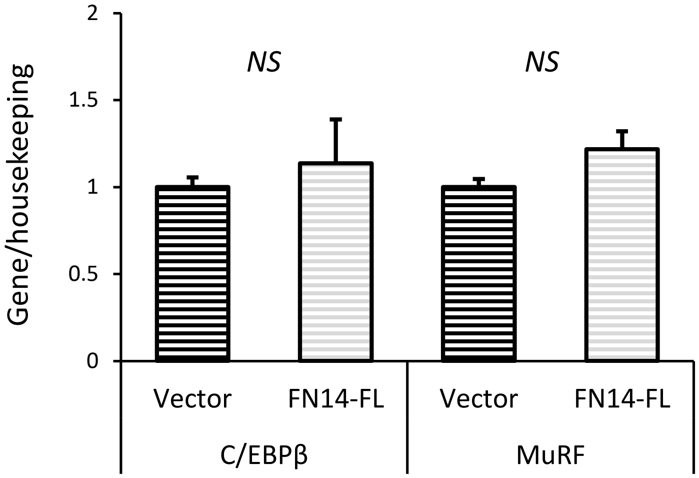

Supplementary figure 6. In human myotubes, plasmid-driven overexpression of full length FN14 (FN14-FL) does not alter C/EBPβ or MuRF gene expression. Paired t-tests were used to analyze data using 2 wells of each treatment in 2 individual cell lines (N=4 data points per condition).

Supplementary table 1. PCR Primers

**Gene**	**Primer sequence**
RNA18S5-forward	TTCGGACGTCTGCCCTATCAA
RNA18S5-reverse	ATGGTAGGCACGGCGACTA
B2M-forward	GATGAGTATGCCTGCCGTGT
B2M-reverse	TGCGGCATCTTCAAACCTCC
PGK1-forward	CTCTGCTGGGCAAGGATGTT
PGK1-reverse	CTCCAGCAGGATGACAGACC
CEBPβ-LAP-forward	CCCGCCCGTGGTGTTATTTA
CEBPβ-LAP-reverse	GCATCAACTTCGAAACCGGC
MuRF-forward	GAGGATTCCCGTCGAGTGAC
MuRF-reverse	CGCTGCAGCAACTCACTTTT
FN14-forward	CTCTGAGCCTGACCTTCGTG
FN14-reverse	GTCTCCTCTATGGGGGTGGT
TWEAK-forward	CGATCGCAGCCCATTATGAAG
TWEAK-reverse	TGTTGATTCTGGCTTCCTCCC

Supplementary Table 2. Subject menopause status and medications

ID	Age	Menopause status	Medications
M100	39	Male	Allergy shots
M101	55	Menopause	Atenolol 25 mg, Levothyroxine 138 µg, Potassium 10 mg, Vitamin D, Hot Flash Herbal
M102	51	Menopause	Zoloft 50 mg
M103	45	Menopause	Metoprolol 25 mg, Levothyroxine 125 µg
M105	64	Menopause	Restasis BID, Doxycyline 100 mg, Loratidine 10 mg, multivitamin, Esther-C 1000 mg, Zinc 30 mg, Cal/Mag/ D liquid, Lecithin 2400 mg, COQ10 100 mg
M106	28	Cycling	Neurontin 300 mg, Imitrex PRN
M107	59	Male	Lisinopril 10 mg
M109	62	Male	Prevastatin 20 mg, topical testosterone
M111	29	Male	None
M113	26	Male	None
M124	43	Cycling	Zyprexa 10 mg
M126	59	Menopause	None
M127	42	Menopause	CQ10, Riboflavin, Magnesium, Levothyroxine 125 µg, Estradiol
M131	61	Male	Cymbalta 60 mg, Vitamin D1, B12
M133	29	Cycling	None
M135	52	Cycling	None
M136	53	Menopause	Singulair, Advair, Flonase
M138	68	Menopause	Levothyroxine 75µg, Wellbutrin 150 mg
M139	61	Menopause	Procardia xl 30 mg, Trazadone 50 mg
M140	33	Cycling	Spironolactone HCTZ 10 mg, Sucralfate 10 mg, Tylenol PM, Benadryl
M141	51	Cycling	Adderall XL 10 mg, Wellbutrin 300 mg, Vitamin D, Zinc, B12
M143	44	Cycling	Lexapro 20 mg, multivitamin, Calcium, Vitamin D
M146	41	Cycling	None

Supplementary table 3. Non-significant relationships between changes in M2 macrophages/fiber and changes in gene expression following endurance training.

Gene name	Alternate name	r	P
**Inflammation**			
CD86	CD86 molecule, B7-2	0.34	0.178
CXCL8	C-X-C motif chemokine ligand 8, IL8	0.33	0.190
MPO	Myeloperoxidase	-0.30	0.234
TNFRSF12A	TNF receptor superfamily member 12A , FN14	-0.30	0.241
IL18	Interleukin 18	-0.29	0.263
F3	Coagulation factor III, tissue factor	0.26	0.322
TNF	Tumor necrosis factor, TNFα	0.26	0.323
CEBPB	CCAAT/enhancer binding protein β	0.24	0.364
IL4	Interleukin 4	-0.23	0.371
CCL17	C-C motif chemokine ligand 17	0.23	0.383
IL12B	Interleukin 12B	-0.22	0.398
IL6	Interleukin 6	-0.21	0.423
CSF2	Colony stimulating factor 2, GMCSF	0.19	0.471
IL1B	Interleukin 1β	0.18	0.482
NOS2	Nitric oxide synthase 2, iNOS	0.17	0.504
CCL2	C-C motif chemokine ligand 2, MCP-1	0.17	0.519
CEBPA	CCAAT/enhancer binding protein α	0.13	0.632
CCL8	C-C motif chemokine ligand 8, MCP-2	0.11	0.675
CD163	CD163 molecule	0.11	0.676
IL15	Interleukin 15	0.05	0.847
**Growth**			
MYOD1	Myogenic differentiation 1	0.47	0.059
WNT10B	Wnt family member 10B	0.41	0.104
MYOG	Myogenin	-0.22	0.403
MSTN	Myostatin, GDF8	0.15	0.556
IGF1R	Insulin like growth factor 1 receptor	0.11	0.682
FGF2	Fibroblast growth factor 2, basic FGF	-0.08	0.756
CNTF	Ciliary neurotrophic factor	0.05	0.844
TRIM63	Tripartite motif-containing 63, MuRF1	0.05	0.863
FBXO32	F-box protein 32, MAFbx, Atrogin-1	0.03	0.899
WNT2	Wnt family member 2	0.01	0.982
**Extracellular matrix**			
MMP2	Matrix metallopeptidase 2	0.48	0.054
TIMP2	TIMP metallopeptidase inhibitor 2	0.47	0.060
MMP9	Matrix metallopeptidase 9	0.39	0.118
TIMP1	TIMP metallopeptidase inhibitor 1	0.18	0.499
CTGF	Connective tissue growth factor	0.05	0.856

M2 (CD11b+/CD206+) macrophages were assessed by immunohistochemistry. Gene expression was quantified using a custom-designed NanoString nCounter system code set. r = Pearson correlation coefficient. P values are not adjusted for multiple comparisons N = 17.

Supplementary table 4. Effect of endurance exercise training on inflammation, growth, and ECM-related gene expression in human *vastus lateralis*.

Gene name	Alternate name	Pre-training (mean ± SEM)	Post-training (mean ± SEM)	P=
**Inflammation**				
CCL8	C-C motif chemokine ligand 8, MCP-2	6.2 ± 0.61	8.3 ± 0.81	**0.029***
NOS2	Nitric oxide synthase 2, iNOS	4.5 ± 0.51	6.6 ± 0.82	**0.043***
ITGAM‡	Integrin subunit alpha M, CD11b	31.9 ± 2.03	39.5 ± 3.06	**0.049***
CCL5‡	C-C motif chemokine ligand 5, RANTES	32.7 ± 3.76	47.1 ± 6.16	0.063
MPO	Myeloperoxidase	5.6 ± 0.55	4.2 ± 0.56	0.065
MRC1‡	Mannose receptor C-type 1, CD206	73.5 ± 5.78	89.4 ± 8.27	0.097
IL18	Interleukin 18	26.2 ± 2.82	32.1 ± 4.01	0.107
CD3D	Cluster of differentiation 3-δ	11.5 ± 1.67	15.9 ± 2.48	0.163
CD163	Cluster of Differentiation 163	48.1 ± 3.91	56.2 ± 5.23	0.176
CD68‡	Cluster of Differentiation 68	29.7 ± 2.63	35.3 ± 3.22	0.182
CSF2	Colony stimulating factor 2, GM-CSF	4.2 ± 0.54	4.9 ± 0.46	0.257
CCL17	C-C motif chemokine ligand 17	4.7 ± 0.75	6.0 ± 0.84	0.270
CCL2	C-C motif chemokine ligand 2, MCP-1	29.1 ± 1.92	32.8 ± 2.70	0.301
CCL18‡	C-C motif chemokine ligand 18	14.1 ± 2.06	18.8 ± 4.03	0.322
ITGAX‡	Integrin subunit alpha X, CD11c	35.69 ± 6.51	45.0 ± 8.18	0.412
CEBPA	CCAAT/enhancer binding protein α	57.9 ± 4.47	60.6 ± 3.27	0.54
CXCL8	C-X-C motif chemokine ligand 8, IL8	5.7 ± 0.98	6.6 ± 1.26	0.620
HMOX1‡	Heme oxygenase 1, HO-1	179.9 ± 44.67	159.3 ± 17.62	0.695
IL12B	Interleukin 12B	6.6 ± 1.07	7.0 ± 0.61	0.790
IL15	Interleukin 15	18.6 ± 1.45	18.9 ± 1.61	0.831
IL1B	Interleukin 1-β	6.10 ± 0.72	6.0 ± 0.79	0.883
F3	coagulation factor III, tissue factor	83.8 ± 6.16	83.1 ± 7.18	0.934
CD86	Cluster of Differentiation 86, B7-2	11.2 ± 1.09	11.2 ± 0.88	0.973
**Growth**				
HGF‡	Hepatocyte growth factor	13.5 ± 1.23	19.4 ± 1.76	**0.005****
MSTN	Myostatin, GDF8	232.6 ± 27.69	165.5 ± 18.8	**0.015***
IGF1‡	Insulin like growth factor 1	61.3 ± 4.07	74.4 ± 5.10	**0.035***
MYOD1	Myogenic differentiation 1	600.8 ± 48.63	531.1 ± 42.35	0.140
FBXO32	F-box protein 32, MAFbx, Atrogin-1	1272.7 ± 111.12	1172.9 ± 78.41	0.302
FGF2	Fibroblast growth factor 2, basic FGF	49.1 ± 4.26	52.5 ± 2.64	0.349
MYOG	Myogenin	5.7 ± 0.59	5.3 ± 0.55	0.502
CNTF	Ciliary neurotrophic factor	41.2 ± 2.30	43.7 ± 4.74	0.666
IGF1R	Insulin like growth factor 1 receptor	141.5 ± 8.38	144.6 ± 5.25	0.707
WNT10B	Wnt family member 10B	6.5 ± 0.73	6.9 ± 0.83	0.732
WNT2	Wnt family member 2	5.2 ± 0.81	5.1 ± 0.70	0.906

Supplementary table 4, continued. Effect of endurance exercise training on inflammation, growth, and ECM-related gene expression in human *vastus lateralis*.

Gene name	Alternate name	Pre-training (mean ± SEM)	Post-training (mean ± SEM)	P=
**Extracellular Matrix**				
SPARC‡	Osteonectin	1359 ± 94.9	2128 ± 197.4	**<0.001*** **
TGFB1‡	Transforming growth factor β-1	61.2 ± 4.98	85.1 ± 5.67	**0.001** **
LOX‡	Lysyl oxidase	16.0 ± 2.70	37.8 ± 4.80	**0.002** **
COL6A1‡	Collagen type VI α-1 chain	380.3 ± 20.42	492.3 ± 38.49	**0.009** **
MMP14	Matrix metallopeptidase 14	33.7 ± 2.72	54.1 ± 6.14	**0.010***
COL5A1‡	Collagen type V α-1 chain	145.8 ± 15.37	224.0 ± 25.88	**0.013***
TIMP2	TIMP metallopeptidase inhibitor 2	332.0 ± 25.23	419.6 ± 32.45	**0.031***
MMP2	Matrix metallopeptidase 2	135.2 ± 8.46	180.7 ± 18.81	**0.032***
SERPINE1‡	Plasminogen activator inhibitor-1, PAI-1	14.6 ± 1.73	19.7 ± 2.17	**0.040***
TIMP1	TIMP metallopeptidase inhibitor 1	112.4 ± 10.39	139.2 ± 9.79	0.054
MMP9	Matrix metallopeptidase 9	5.8 ± 0.89	8.3 ± 1.65	0.165
ELN‡	Elastin	34.1 ± 6.79	47.7 ± 7.40	0.196
CTGF	Connective tissue growth factor	118.7 ± 26.20	126.2 ± 13.67	0.814

Gene expression was measured with a custom-designed NanoString nCounter system code set. Pre- and post-training mRNA counts were compared using paired t-tests. ‡Change in gene expression was also correlated to change in M2 macrophages/fiber (see Figure 4 and Table 2). ***P < 0.001, **P <0.01, *P < 0.05. P values are not adjusted for multiple comparisons. N = 20.
